# Efficacy and safety of febuxostat extended release and immediate release in patients with gout and moderate renal impairment: phase II placebo-controlled study

**DOI:** 10.1186/s13075-018-1593-0

**Published:** 2018-05-30

**Authors:** Lhanoo Gunawardhana, Michael A. Becker, Andrew Whelton, Barbara Hunt, Majin Castillo, Kenneth Saag

**Affiliations:** 1 Takeda Pharmaceuticals, One Takeda Parkway, Deerfield, IL 60015 USA; 2 University of Chicago Pritzker School of Medicine, 5841 S. Maryland Avenue, Chicago, IL 60637 USA; 3 Johns Hopkins University, 1737 Beaver Brook Lane, Hunt Valley, MD 21030 USA; 4 Birmingham VA Medical Center, 700 S. 19th Street, Birmingham, AL 35233 USA; 5 University of Alabama at Birmingham, Faculty Office Tower, Suite 820, 1720 2nd Avenue South, Birmingham, AL 35294 USA

**Keywords:** Hyperuricemia, Renal impairment, Febuxostat, Extended release, Serum uric acid

## Abstract

**Background:**

Febuxostat immediate release (IR), a xanthine oxidase inhibitor, is indicated for the management of hyperuricemia in patients with gout by lowering urate levels. An extended release (XR) formulation of febuxostat was developed to provide equal or superior efficacy on urate lowering compared with the IR formulation and potentially lower the risk of treatment-initiated gout flares due to an altered pattern of drug exposure. The present study evaluated the efficacy and safety of febuxostat XR and IR formulations in patients with gout and moderate renal impairment (estimated glomerular filtrate rate ≥ 30 and < 60 ml/min).

**Methods:**

This was an exploratory, 3-month, phase II, multicenter, placebo-controlled, double-blind proof-of-concept study. Patients (*n* = 189) were randomized 1:1:1:1:1 to receive placebo or febuxostat IR 40 mg, XR 40 mg, IR 80 mg, or XR 80 mg once daily. Endpoints included: proportion of patients with serum uric acid (sUA) < 5.0 mg/dl at month 3 (primary endpoint), proportion of patients with sUA < 6.0 mg/dl at month 3, and proportion of patients with ≥ 1 gout flare requiring treatment over 3 months.

**Results:**

At month 3, all febuxostat treatment groups were associated with greater proportions of patients achieving sUA < 5.0 mg/dl (*p* < 0.05 vs placebo). A greater proportion of patients receiving XR 40 mg achieved sUA < 5.0 mg/dl versus those receiving IR 40 mg (*p* = 0.034); proportions were similar in the IR 80 mg and XR 80 mg groups. Higher proportions of febuxostat-treated patients achieved sUA < 6.0 mg/dl at month 3 (*p* < 0.05 vs placebo) and experienced ≥ 1 gout flare (significant for all comparisons, except XR 40 mg). Incidences of treatment-related adverse events were low across all treatment groups; the majority were mild or moderate with no apparent trends correlating with IR or XR doses. The most common treatment-emergent adverse event was hypertension. One death (unrelated to the study drug) was reported.

**Conclusions:**

These exploratory data demonstrate that febuxostat (XR and IR) formulations were effective and well tolerated in patients with gout and moderate renal impairment.

**Trial registration:**

ClinicalTrials.gov, NCT02128490 Registered on 29 April 2014.

**Electronic supplementary material:**

The online version of this article (10.1186/s13075-018-1593-0) contains supplementary material, which is available to authorized users.

## Background

The primary etiology of gout is considered to be hyperuricemia; however, gout is also associated with a substantial burden of comorbidities. In particular, hyperuricemia is strongly associated with the risk of renal disease [1, 2]. Conversely, renal impairment is recognized as a significant risk factor for gout [2–6]. Over 70% (~ 5.5 million) of US adults with both gout and hyperuricemia also have chronic kidney disease (CKD) stage ≥ 2 (estimated glomerular filtration rate (eGFR) < 90 ml/min/1.73 m^2^) [2]. Moreover, increasing levels of hyperuricemia are associated with a graded increase in the prevalence of CKD. In US patients with serum uric acid (sUA) levels ≥10 mg/dl, the prevalence of CKD stage ≥ 2 is > 85% [2].

Xanthine oxidase (XO) inhibitors are the first-line pharmacologic agents recommended in the USA for urate-lowering therapy (ULT) in the management of chronic gout. In the USA, febuxostat and allopurinol are the only XO inhibitors indicated for the management of hyperuricemia (defined as sUA exceeding the limit of solubility (~ 6.8 mg/dl) at core body temperature) in patients with gout [1, 7]. Both febuxostat and allopurinol exert their urate-lowering effect by inhibiting the enzyme XO, which catalyzes the synthesis of uric acid from its precursors, hypoxanthine and xanthine. However, allopurinol and febuxostat differ significantly in how they are metabolized and eliminated from the body. Allopurinol and its primary active metabolite, oxypurinol, are primarily eliminated via the kidneys. While allopurinol is relatively well tolerated and effective in patients with gout [8], its elimination via the kidneys may complicate its use in patients with gout and impaired renal function. However, recent studies evaluating dose escalation reported that higher allopurinol doses were effective and well tolerated in this setting [9].

Unlike allopurinol, febuxostat has a relatively low renal excretion. Moreover, clearance of febuxostat by hepatobiliary conjugation is unaltered in the presence of renal impairment [10, 11]. The efficacy and safety of immediate-release (IR) 40 mg and 80 mg febuxostat once daily (QD) in managing hyperuricemia in gout patients has been demonstrated in several randomized controlled trials involving patients with normal renal function, and patients with mild-to-moderate renal impairment [10, 12–16]. The US label for febuxostat indicates that it can be used in patients with mild-to-moderate renal impairment without the need for dose adjustments [11]. This article provides additional efficacy and safety data for the approved febuxostat IR formulation and an extended-release (XR) formulation of febuxostat in patients with moderate renal impairment, a segment of the gout population with significant unmet medical needs with regards to ULT.

The XR febuxostat formulation was developed with the aim of providing equal or greater reductions in sUA levels compared with the IR formulation, with reduced incidence of treatment-initiated flares due to an altered pattern of drug exposure. Pharmacokinetic analysis in a phase I trial demonstrated that prototypes of the XR formulation were associated with a lower and more stable febuxostat exposure (reduced area under the plasma concentration–time curve and maximum observed plasma concentration) compared with the IR formulation [17]. It was thus postulated that the more stable drug exposure and reduced daily variability in urate levels associated with febuxostat XR would reduce the likelihood of activation of inflammation in joints due to mobilization of urate crystals and subsequent gout flares. Here, we present results from a 3-month, phase II, proof-of-concept study conducted to evaluate the efficacy and safety of febuxostat IR and XR in patients with gout and moderate renal impairment.

## Methods

### Study design

**Fig. 1:**
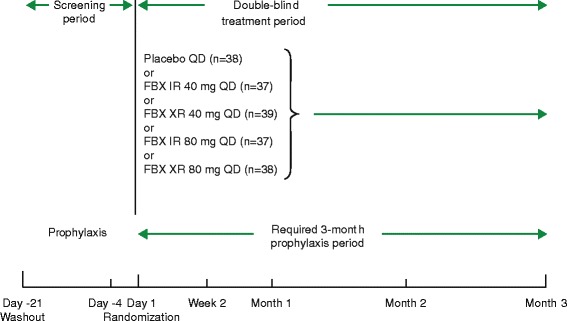
Study design. FBX febuxostat, IR immediate release, QD once daily, XR extended release

NCT02128490 was a phase II, multicenter, randomized, double-blind, placebo-controlled, exploratory study involving patients at 64 sites across the USA from May 5, 2014 to October 23, 2015. The study design consisted of a 3-week screening/washout period, followed by a 3-month double-blind treatment period (Fig. 1). Patients receiving ULT at enrollment discontinued their medication on the day −21 screening visit; this was followed by a 3-week washout period (day −21 to day −1). A day −4 visit served as a follow-up screening visit for patients washing out ULT, and as the initial screening visit for patients not receiving ULT. All patients had their blood drawn at the day −4 screening visit to determine baseline sUA levels. Patients who did not meet day −4 sUA criteria were allowed to have one retest of the sUA levels at the discretion of the investigator. Patients who met all eligibility criteria received the double-blind study drug from day 1 through the 3-month study duration. Eligible patients underwent physical examinations, clinical assessments, review of concomitant medication usage, and an electrocardiogram (ECG) on day 1. Follow-up visits were scheduled at week 2, month 1, month 2, and month 3 (end of treatment). If applicable, an ECG, urinalysis, and a pregnancy test were performed at the end-of-treatment visit. Each investigator conducted the study according to the Declaration of Helsinki and the International Conference on Harmonisation’s Harmonised Tripartite Guideline for good clinical practices, and in compliance with applicable local or regional regulatory requirements. All patients enrolled in the study provided written informed consent in the manner deemed appropriate by the institutional review board or the independent ethics committees.

### Patients

Eligible patients included men and women (aged ≥ 18 years) who: provided informed consent; had a history or presence of gout based on criteria defined by the American Rheumatism Association [18]; had an sUA level ≥ 8.0 mg/dl at the day −4 screening visit or at the retest visit; had moderate renal impairment as defined by an eGFR (Modification of Diet in Renal Disease) ≥ 30 and < 60 ml/min at screening visit on day −21 for patients on ULT and on day −4 for patients not on ULT or at the retest visit; and had a self-reported history of ≥ 1 gout flare within the 12 months prior to the screening visit.

Patients were excluded from the study if they met any of the following criteria: received an investigational compound within 30 days prior to screening, secondary hyperuricemia; history of xanthuria, known hypersensitivity to febuxostat or any components in its formulations, known hypersensitivity to naproxen, any other nonsteroidal anti-inflammatory drug (NSAID), aspirin, lansoprazole, colchicine, or any components in their formulation; had experienced either a myocardial infarction, stroke, hospitalized unstable angina, cardiac or cerebrovascular revascularization procedure, or hospitalized transient ischemic attack; or had a history of cancer (other than basal cell carcinoma of the skin) within 5 years prior to the screening visit, history of drug or alcohol abuse, presence of rheumatoid arthritis, active peptic ulcer disease, or any significant medical condition that would interfere with the treatment, safety, or compliance with the protocol.

### Randomization

Eligible patients were randomly assigned in a 1:1:1:1:1 ratio to one of five treatment groups: placebo, febuxostat IR 40 or 80 mg, or febuxostat XR 40 or 80 mg, all QD. Patients were randomized within two population strata: patients with an sUA level ≥ 8.0 and < 10.0 mg/dl at the day −4 visit; and patients with an sUA level ≥ 10.0 mg/dl at the day −4 visit. An interactive voice or web response technology was employed for randomization, and for assigning the study drug.

### Treatments

Patients orally self-administered one capsule of the study drug starting from day 1 through the end of study duration (month 3). To maintain blinding, all tablets (febuxostat XR, febuxostat IR, and placebo) were overencapsulated in a similar manner. To maintain consistency, patients were instructed to take the study drug at the same time daily, and to withhold the study drug on days of scheduled follow-up visits (months 1, 2, and/or 3) when samples for trough pharmacokinetic measurements were collected. All patients systematically received gout flare prophylaxis for the duration of double-blind treatment from day 1 to the end of treatment, including colchicine 0.6 mg every other day (QOD); however, if colchicine was contraindicated or not tolerated, naproxen (250 mg BID) or other NSAIDs or prednisone were permitted at the investigator’s discretion. Beginning on the day −21 screening visit, patients receiving ULT discontinued their ULT and entered a washout period during which they received 0.6 mg colchicine QOD for gout flare prophylaxis. From day 1 through to end of treatment, all remaining patients (i.e., those not receiving ULT prior to the study) also began treatment for gout flare prophylaxis.

### Study endpoints

The primary efficacy endpoint was percentage of patients with sUA < 5.0 mg/dl at month 3. Secondary efficacy endpoints included the proportion of patients with sUA < 6.0 mg/dl at month 3, and the proportion of patients with ≥ 1 gout flare requiring treatment during the 3-month treatment period.

### Safety assessments

Safety and tolerability were assessed by evaluating the incidence of treatment-emergent adverse events (TEAEs), 12-lead ECG findings, clinical laboratory tests, and vital signs. All safety analyses were conducted using the safety analysis set, which included all patients who received ≥ 1 dose of double-blind study drug. A TEAE was defined as any AE, regardless of relationship to the study drug, which occurred on or after the first double-blind dose date and up to 30 days after the last dose date of the double-blind study drug. A pretreatment event was defined as an event with a reported onset date after the patient signed the consent form but prior to the date of randomization. Treatment-related AEs were defined as TEAEs that were identified by the investigator to be related to the study drug. TEAEs were identified as reported by the investigators and summarized using the MedDRA Version 18.0 coding dictionary.

### Statistical analyses

Primary efficacy analyses were performed using the full analysis set, and included all patients who received ≥ 1 dose of the double-blind study drug. For the primary analysis, no missing data were imputed. The original protocol was modified (prior to database lock) so that patients who discontinued treatment before the month 3 visit were included in the efficacy analysis as having failed to meet a particular efficacy endpoint. sUA levels obtained > 1 day after the last dose of double-blind study drug were excluded from the analyses.

Efficacy analysis primarily involved comparison of treatment with febuxostat XR 40 and 80 mg QD versus febuxostat IR 40 and 80 mg QD, respectively. In addition, each febuxostat XR treatment group and the placebo group were compared by study visit. Comparisons were also made between febuxostat XR 40 and 80 mg QD, between febuxostat IR 40 and 80 mg QD, and between each febuxostat IR treatment group and placebo.

A gout flare was defined, in agreement with the US Food and Drug Administration, as the presence of all of the following: patient-reported acute articular pain typical of a gout attack that was deemed (by patient and/or investigator) to require treatment (and was treated) with colchicine, NSAIDs, or steroids; presence of at least three or more of joint swelling, redness, tenderness, and pain; and patient experienced at least one or more of rapid onset of pain, decreased range of motion, joint warmth, and other symptoms similar to a prior gout flare. Patients who discontinued the double-blind study drug due to gout flares were considered to have experienced a gout flare during the 3-month treatment period.

The SAS System with the HP-Unix operating system was used to perform the statistical analyses. All statistical tests and confidence intervals (CIs) were two sided and conducted at the 0.05 significance level, with no adjustments for multiple comparisons. Fisher’s exact test was used to compare treatment groups for the analysis of primary and secondary efficacy endpoints. A total of 200 patients (40 per treatment group) were planned for enrollment into this study. Due to the exploratory nature of the proof-of-concept study, the sample size was not based on formal power calculations. The sample size was considered sufficient to characterize the effect of febuxostat XR formulation on lowering of urate levels compared with the febuxostat IR formulation.

## Results

### Disposition of study patients

**Fig. 2:**
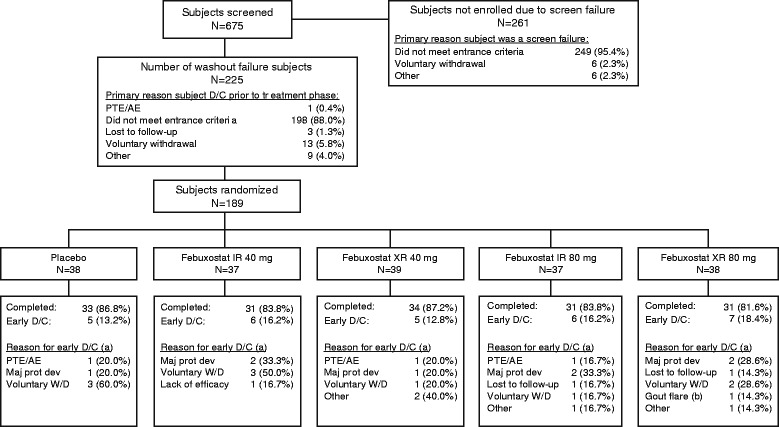
Disposition of patients. (a) Percentage is based on number of patients with early D/C for the treatment group. (b) If a patient had a gout flare that caused W/D from the study, it was recorded as ‘Wishes to withdraw from study drug due to gout flare’ instead of adverse event. Note: 38.7% (261 patients) were excluded at screening, and 33.3% (225 patients) were excluded at washout. Most patients who were not enrolled (249/261, 95.4%) had failed to meet the screening criteria. AE adverse event, D/C discontinuation of study drug, IR immediate release, Maj prot dev major protocol deviation, PTE pretreatment event, W/D withdrawal, XR extended release

Of the 675 patients screened, 189 (28%) were randomized to receive one of five treatments (placebo, *n* = 38; febuxostat IR 40 mg, *n* = 37; febuxostat XR 40 mg, *n* = 39; febuxostat IR 80 mg, *n* = 37; febuxostat XR 80 mg, *n* = 38; Fig. 2). Of these patients, 261 (39%) were not enrolled due to screening failure and 225 (33%) were not enrolled due to washout failure; the primary reason in both instances was failure to meet the entry criteria (249/261, 95% and 198/225, 88%, respectively). Overall, 29/189 (15.3%) of patients discontinued the study drug early; the most common reasons for early discontinuation were voluntary withdrawal (10/29, 34.5%) and major protocol deviation (7/29, 24.1%). The percentage of early discontinuations was lowest in the febuxostat XR 40 mg group (5/39, 12.8%) and highest in the febuxostat XR 80 mg group (7/38, 18.4%). Mean treatment compliance ((capsules dispensed minus capsules returned)/(days on drug) × 100%) ranged from 93.5 to 98.3% across treatment groups. All randomized patients were included in both the full analysis and safety analysis data sets.

### Demographics and baseline characteristics

Demographic and baseline characteristics for patients in the treatment groups are summarized in Table 1. Overall, demographic characteristics were similar across treatment groups at baseline. The study population was predominantly male (70.9%) and white (66.7%); mean age was 63.1 years (range 22–87 years) and mean (standard deviation) (SD) body mass index (BMI) was 34.3 (7.8) kg/m^2^. The overall mean (SD) sUA at baseline was 9.71 (1.25) mg/dl; two-thirds of patients (66.7%, 126/189) had baseline sUA ≥ 9.0 mg/dl. Gout and renal baseline characteristics were balanced across the treatment groups. Overall, approximately 88% of patients had experienced a gout flare within the 6 months prior to study entry date, and most patients (72%) had previously received a ULT.

**Table 1:** Demographic and patient characteristics at baseline

Variable	Placebo (*n* = 38)	FBX IR 40 mg (*n* = 37)	FBX XR 40 mg (*n* = 39)	FBX IR 80 mg (n = 37)	FBX XR 80 mg (*n* = 38)
Age (years), mean (SD)	64.6 (12.8)	61.3 (10.1)	64.4 (11.2)	63.5 (10.3)	61.4 (12.2)
Sex, *n* (%)					
Male	26 (68.4)	25 (67.6)	26 (66.7)	28 (75.7)	29 (76.3)
Female	12 (31.6)	12 (32.4)	13 (33.3)	9 (24.3)	9 (23.7)
Race, *n* (%)^a^					
White	33 (86.8)	24 (64.9)	26 (66.7)	21 (56.8)	22 (57.9)
Black or African American	4 (10.5)	10 (27.0)	10 (25.6)	12 (32.4)	10 (26.3)
BMI (kg/m^2^), mean (SD)	34.1 (7.0)	36.5 (9.0)	35.2 (8.7)	33.1 (7.2)	32.6 (6.6)
Baseline sUA (mg/dl), mean (SD)^b^	9.7 (1.2)	9.8 (1.4)	9.6 (1.3)	9.6 (1.1)	9.8 (1.4)
Approximate gout flares during past year, *n* (%)^b^					
1–3	24 (63.2)	22 (59.5)	27 (69.2)	23 (62.2)	22 (57.9)
4–6	10 (26.3)	6 (16.2)	9 (23.1)	9 (24.3)	9 (23.7)
> 6	4 (10.5)	9 (24.3)	3 (7.7)	5 (13.5)	7 (18.4)
Baseline eGFR (ml/min), mean (SD)^c^	47.3 (9.4)	46.0 (8.3)	43.3 (6.7)	48.2 (7.5)	48.3 (8.8)

Data from the full analysis set unless indicated otherwise

*BMI* body mass index, *eGFR* estimated glomerular filtration rate, *FBX* febuxostat, *IR* immediate release, *SD* standard deviation, *sUA* serum uric acid, *XR* extended release

^a^Total numbers (%) of patients classified as ‘Asian’, ‘Native Hawaiian or Other Pacific Islander’, and ‘Other’ were 9 (4.8), 4 (2.1), and 4 (2.1), respectively

^b^Data from the safety analysis set (*n* same as full analysis set)

^c^All patients had moderately impaired renal function at baseline, defined as between ≥ 30 ml/min and < 60 ml/min, in line with study inclusion criteria

### Efficacy

#### Primary efficacy endpoint

**Fig. 3:**
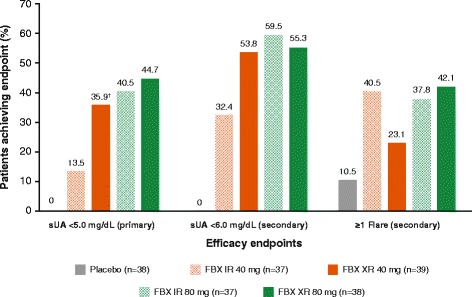
Percentage of patients who achieved primary and secondary outcomes (full analysis set^*^). *p* < 0.05 versus placebo for all active treatment groups for all three endpoints, with the exception of ‘≥ 1 flare’, where there was no statistically significant difference between FBX XR 40 mg and placebo. ^†^*p* < 0.05 versus equivalent-dose IR formulation. FBX febuxostat, IR immediate release, sUA serum uric acid, XR extended release

Figure 3 shows the percentage of patients achieving the primary endpoint of sUA < 5.0 mg/dl at the month 3 visit. Both formulations and doses of febuxostat were associated with patients achieving sUA < 5.0 mg/dl at month 3 (13.5–44.7%; *p* = 0.03 for IR 40 mg vs placebo and *p* < 0.001 for all other comparisons). The proportion of patients achieving sUA < 5.0 mg/dl at month 3 was significantly higher among those receiving febuxostat XR 40 mg compared with those receiving febuxostat IR 40 mg (35.9% vs 13.5%; *p* = 0.03). At the month 3 visit, a similar proportion of patients treated with febuxostat XR 80 mg achieved the primary endpoint compared with IR 80 mg (*p* = 0.82).

#### Secondary efficacy endpoints

Febuxostat treatment was associated with patients achieving sUA < 6.0 mg/dl at month 3 versus placebo (32.4–59.5%; *p* < 0.001) (Fig. 3), including both the 40 and 80 mg doses of the XR formulation. A numerically greater proportion of patients treated with febuxostat XR 40 mg achieved sUA < 6.0 mg/dl at month 3 compared with patients treated with IR 40 mg; similar proportions of patients receiving febuxostat XR 80 mg and IR 80 mg achieved this endpoint.

With the exception of the XR 40 mg dose, febuxostat treatment was associated with significantly greater proportions of patients with ≥ 1 gout flare compared with placebo (Fig. 3). A numerically smaller proportion of patients treated with febuxostat XR 40 mg experienced flares compared with the IR 40 mg dose group (23.1% vs 40.5%, respectively; *p* = 0.14), although no significant differences were noted for febuxostat XR 80 mg versus IR 80 mg formulation (*p* = 0.82).

#### Safety and tolerability

Overall, 36.5% (69/189) of patients reported experiencing ≥ 1 TEAE. The incidences of treatment-related TEAEs (11/189, 5.8%) and serious TEAEs (6/189, 3.2%) were low across all treatment groups. The majority of TEAEs (126/133, 94.7%) were mild or moderate in intensity, and there were no apparent trends observed with respect to the dose level or formulation. Table 2 presents an overview of TEAEs, treatment-related TEAEs, and serious TEAEs.

**Table 2:** Overview of TEAEs, treatment-related TEAEs, and serious TEAEs

	Patients experiencing any TEAE, *n* (%)				
	Placebo (*n* = 38)	FBX IR 40 mg (*n* = 37)	FBX XR 40 mg (*n* = 39)	FBX IR 80 mg (n = 37)	FBX XR 80 mg (*n* = 38)
Overall TEAEs	13 (34.2)	16 (43.2)	16 (41.0)	11 (29.7)	13 (34.2)
Related to treatment	2 (5.3)	4 (10.8)	2 (5.1)	1 (2.7)	2 (5.3)
Not related to treatment	11 (28.9)	12 (32.4)	14 (35.9)	10 (27.0)	11 (28.9)
TEAEs by severity					
Mild	7 (18.4)	8 (21.6)	9 (23.1)	6 (16.2)	4 (10.5)
Moderate	6 (15.8)	7 (18.9)	6 (15.4)	5 (13.5)	7 (18.4)
Severe	0	1 (2.7)	1 (2.6)	0	2 (5.3)
TEAEs leading to study drug discontinuation	2 (5.3)	0	1 (2.6)	1 (2.7)	0
Serious TEAEs	0	1 (2.7)	1 (2.6)	0	4 (10.5)
Related to treatment	0	0	0	0	0
Not related to treatment^a^	0	1 (2.7)	1 (2.6)	0	4 (10.5)
Leading to study drug discontinuation	0	0	0	0	0
Deaths	0	0	0	0	1 (2.6)

Safety analysis set. ^a^Serious TEAEs reported in each of the treatment groups: FBX IR 40 mg, one patient had both gastroenteritis and acute kidney injury; FBX XR 40 mg, one patient had cholelithiasis; FBX XR 80 mg, three patients had coronary artery disease, gangrene, or hypertension and one patient had both sinus node dysfunction and a fatal cardiac arrest

*FBX* febuxostat, *IR* immediate release, *TEAE* treatment-emergent adverse event, *XR* extended release

The most frequently reported TEAEs (≥ 5% of patients in any treatment group) are summarized in Table 3. The most common TEAE was hypertension. No cases of renal failure (based on renal insufficiency or worsening renal insufficiency) were reported in any of the febuxostat XR or IR treatment groups; however, one case of acute kidney injury was reported in a patient treated with febuxostat IR 40 mg. In contrast, the incidence of renal failure in the placebo group was 5.3% (2/38). There was a low overall incidence of TEAEs considered by the investigator to be treatment related.

**Table 3:** Most common TEAEs (reported by ≥ 5% of patients in any treatment group)

	Patients reporting any TEAEs (by system organ class/preferred term), *n* (%)				
	Placebo (*n* = 38)	FBX IR 40 mg (*n* = 37)	FBX XR 40 mg (*n* = 39)	FBX IR 80 mg (*n* = 37)	FBX XR 80 mg (*n* = 38)
Cardiac disorders					
Palpitations	0	0	2 (5.1)	0	0
Gastrointestinal disorders					
Diarrhea	0	2 (5.4)	0	0	0
Infections and infestations					
Gastroenteritis	0	2 (5.4)	0	0	0
Urinary tract infection	0	2 (5.4)	0	0	0
Metabolism and nutrition disorders					
Hyperglycemia	0	0	2 (5.1)	0	0
Renal and urinary disorders					
Renal failure	2 (5.3)	0	0	0	0
Vascular disorders					
Hypertension	1 (2.6)	1 (2.7)	0	1 (2.7)	4 (10.5)

Safety analysis set

*FBX* febuxostat, *IR* immediate release, *TEAE* treatment-emergent adverse event, *XR* extended release

A total of eight serious TEAEs were reported in six patients in the febuxostat groups; none of these serious TEAEs were considered related to the study drug (Table 2). Serious TEAEs included one death due to acute cardiac event in a patient in the febuxostat XR 80 mg group; this patient also experienced sinus node dysfunction as a nonfatal serious TEAE and had a history of smoking, alcohol consumption, hypertension, diabetes mellitus, heart murmur, hypercholesterolemia, and peripheral edema, and a BMI of 31.3. The remaining six nonfatal serious TEAEs were as follows (by treatment group): febuxostat IR 40 mg, one patient had both gastroenteritis and acute kidney injury; febuxostat XR 40 mg, one patient had cholelithiasis; and febuxostat XR 80 mg, three patients had coronary artery disease, gangrene, or hypertension (medical histories of patients with nonfatal serious TEAEs are summarized in Additional file 1).

In all, four patients (2.1%) experienced a TEAE that resulted in premature discontinuation of a study drug: renal failure or rash macular were reported in two patients in the placebo group; and one patient each in the febuxostat XR 40 mg and IR 80 mg groups experienced abdominal pain and hyperkalemia, respectively, which were assessed by the investigator as related to the study drug.

## Discussion

Febuxostat XR formulations were developed to test the concept that their administration would result in equivalent or greater urate-lowering efficacy compared with the IR formulation, with lower incidences of treatment-initiated flares due to an altered pattern of drug exposure. These potential benefits of febuxostat XR preparations were suggested by the results of two previous studies. The first was a phase I study which demonstrated that prototypes of the febuxostat XR formulation were associated with a reduced and more stable exposure to febuxostat (reduced area under the plasma concentration–time curve and maximum observed plasma concentration) compared with the IR formulation [17]. It was postulated that the more stable drug exposure and reduced daily variability in urate levels associated with febuxostat XR would reduce the likelihood of urate crystal activation of inflammation and subsequent gout flares. The second study was a phase II proof-of-concept trial which demonstrated that febuxostat IR 30 mg BID (designed to mimic the effect of an XR 80 mg dose) and febuxostat IR 40/80 mg QD were both significantly more effective at reducing sUA than placebo in patients with moderate-to-severe renal impairment [7]. The current randomized, placebo-controlled, double-blind, phase II study was designed to evaluate the efficacy and tolerability of currently approved doses of febuxostat IR (40 and 80 mg QD) compared with the same doses in an XR formulation. In addition, a placebo arm was included to allow the comparison of the safety and efficacy of both formulations of febuxostat versus placebo, particularly in the setting of moderate renal impairment.

Two key findings emerged from the results of this proof-of-concept study. Firstly, both the XR and IR formulations of febuxostat were associated with reductions in sUA to both the < 5 mg/dl and < 6 mg/dl target levels in this population of patients with moderate impairment of renal function. Secondly, no meaningful differences were reported in TEAEs across treatment arms, indicating that both formulations of febuxostat (XR and IR) were well tolerated in patients with this level of renal impairment. In terms of TEAEs of special interest, there was one case of acute kidney injury with febuxostat treatment (IR 40 mg) and two cases of renal failure (based on renal insufficiency) with placebo.

In addition, our data suggest that there were statistically significant treatment benefits in favor of febuxostat XR 40 mg versus IR 40 mg in terms of the proportions of patients achieving the target sUA levels (< 5 mg/dl and < 6 mg/dl), with numerical trends toward reductions in the proportions of patients experiencing gout flares ( significant). We were, however, unable to confirm superior efficacy (in terms of the primary and secondary endpoints) of febuxostat XR versus IR at doses of 80 mg. Moreover, efficacy data from a follow-up phase III study, which involved a similar patient population and study design, suggest no significant treatment benefits in favor of febuxostat XR 80 mg versus IR 80 mg [19].

Nevertheless, the results of this study reinforce the findings reported in other short-term and long-term studies that suggest treatment with febuxostat is well tolerated and effective in the management of hyperuricemia in gout patients with renal impairment. In a recent placebo-controlled exploratory study involving patients with gout who have moderate-to-severe renal impairment (eGFR 15–50 ml/min/1.73 m^2^), patients randomly assigned to receive febuxostat (30 mg febuxostat IR BID or 40/80 mg febuxostat IR QD) demonstrated significantly lower sUA levels and experienced no significant deterioration in renal function. The BID dose of 30 mg febuxostat IR used in this proof-of-concept study was designed to mimic the effect of an XR 80 mg dose [7].

In the 5-year Febuxostat Open-label Clinical trial of Urate-lowering efficacy and Safety (FOCUS) study in patients with gout, febuxostat (IR 40, 80, or 120 mg) was shown to be well tolerated and had a urate-lowering effect that was comparable between patients with normal and impaired renal function (defined as serum creatinine > 1.5 mg/dl or creatinine clearance ≤ 80 ml/min) [16]. Subanalyses of efficacy results from another long-term, open-label trial (Febuxostat/Allopurinol Comparative Extension Long-Term (EXCEL) study) indicated that patients receiving febuxostat (IR 80 or 120 mg) for up to 48 months consistently achieved ~ 50% reductions in baseline sUA levels [20]. Post hoc analyses of the long-term effects of febuxostat in the EXCEL and FOCUS studies also suggested that sustained reductions in sUA to lower concentrations were associated with reductions in the decline of renal function [20], or even stabilization of renal function [21]. Post hoc analyses predicted that every 1 mg/dl of sustained reduction of sUA could potentially result in preserving 1.15 ml/min (EXCEL) [20] or 1 ml/min of eGFR (FOCUS) [21].

Administration of febuxostat appears to produce no apparent adverse effects on renal tubular function, and may also contribute to preserving renal function by protecting the kidney from tissue and vascular injuries resulting from oxidative stress [10, 20]. These findings are corroborated by recent studies in which febuxostat treatment was associated with slower eGFR decline in patients with hyperuricemia who have CKD [22], improvement in endothelial dysfunction and reduced oxidative stress in hemodialysis patients [23], and an overall renoprotective effect in patients with CKD [24].

The Confirmation of Febuxostat in Reducing and Maintaining Serum Urate (CONFIRMS) trial, which included a majority of patients (65%) with mild and moderate renal impairment, demonstrated that the safety profile of febuxostat IR at both 80 mg and 40 mg QD was comparable in patients with mild or moderate renal impairment and those with normal renal function [14]. The present study provides additional data in support of the efficacy and tolerability of febuxostat in patients with moderate renal impairment and its potential to address the unmet need for the effective management of hyperuricemia in this patient population.

The randomized, phase II design of our study was associated with several benefits and limitations. The study design included the use of concurrent control treatment arms to evaluate the efficacy and safety of febuxostat XR against placebo and active comparators (febuxostat IR) [25]. Since the sample size in the present study was not based on formal power calculations, we consider our findings from this trial to be exploratory [26]. A small sample size is known to be associated with greater variability in the data set—a limitation that cannot be excluded for the present study. It is worth noting that a longer study timeframe (> 3 months) may have been more useful for the observation of any potential reductions in the incidence of gout flares in patients undergoing ULT, as these flares are more frequent during the initiation of ULT. In addition, the use of gout flare prophylaxis in this study may have limited detection of a meaningful reduction in incidence of treatment-initiated gout flares. It should be noted that the efficacy and safety of febuxostat IR and XR have also been investigated in a phase III placebo-controlled study in patients with gout and normal or mild-to-severely impaired renal function. In that larger study, both febuxostat formulations were effective in reducing sUA levels to the subsaturation targets (sUA < 5.0 mg/dl and < 6.0 mg/dl) and were well tolerated [19].

## Conclusions

The results of this phase II study support prior reports on the safety and efficacy of febuxostat in patients with gout and moderate renal impairment, and suggest that both the XR and IR formulations of febuxostat are effective and well tolerated in patients with gout and moderately impaired renal function.

## Additional files

Additional file 1: **Table S1**. Summary of medical histories of patients with nonfatal serious TEAEs. (DOCX 21 kb)

Additional file 2: Institutional review boards and reference numbers for the sites in this study. (XLSX 52 kb)

## Abbreviations

BID: Twice daily
BMI: Body mass index
CI: Confidence interval
CKD: Chronic kidney disease
CONFIRMS: Confirmation of Febuxostat in Reducing and Maintaining Serum Urate
ECG: Electrocardiogram
eGFR: Estimated glomerular filtration rate
EXCEL: Febuxostat/Allopurinol Comparative Extension Long-Term
FOCUS: Febuxostat Open-label Clinical trial of Urate-lowering efficacy and Safety
IR: Immediate release
NSAID: Nonsteroidal anti-inflammatory drug
QD: Once daily
QOD: Every other day
SD: Standard deviation
sUA: Serum uric acid
TEAE: Treatment-emergent adverse event
ULT: Urate-lowering therapy
XO: Xanthine oxidase
XR: Extended release
