# Supplementary material for: Efficacy and safety of febuxostat extended release and immediate release in patients with gout and moderate renal impairment: phase II placebo-controlled study
Source: Arthritis Res Ther. 2018 May 30;20:99. doi: 10.1186/s13075-018-1593-0 (PMC5977466; doi:10.1186/s13075-018-1593-0)
Supplement: Supplementary file 1 — Table S1. Summary of medical histories of patients with nonfatal serious TEAEs. (DOCX 21 kb) [file 13075_2018_1593_MOESM1_ESM.docx]

**Table S1:** Summary of medical histories of patients with non-fatal serious TEAEs

| Treatment group/dose | Non-fatal serious TEAEs | Medical history |
| --- | --- | --- |
| FBX IR 40 mg | 1 patient had both gastroenteritis and acute kidney injury | Diabetes mellitus, intermittent hematuria, short gut syndrome, hypertension, metabolic acidosis, ulcerative colitis, ileostomy and a BMI of 36.0 |
| FBX XR 40 mg | 1 patient had cholelithiasis | Diabetes mellitus, hyperlipidemia, hypertension, chronic kidney disease and a BMI of 25.3 |
| FBX XR 80 mg | 1 patient had coronary artery disease | History of smoking, hypertension, chest pain and a BMI of 25.3 |
|  | 1 patient had gangrene | Diabetes mellitus, diabetic foot ulcer, peripheral vascular disease, chronic skin ulcer, diabetic peripheral neuropathy, reconstructive surgery, foot infection and a BMI of 36.9 |
|  | 1 patient had hypertension | Hypertension, diabetes mellitus, hyperlipidemia, chronic venous stasis, cardioaortic and central pulmonary artery enlargement and a BMI of 47.5 |

The medical history for one patient in the FBX XR 80 mg that had both a fatal cardiac arrest and a serious TEAE of sinus node dysfunction is described in the safety section.

BMI = body mass index; FBX = febuxostat; IR = immediate release; TEAE = treatment-emergent adverse event; XR = extended release.
